# Generation of Monocyte-Derived Insulin-Producing Cells from Non-Human Primates According to an Optimized Protocol for the Generation ofPCMO-Derived Insulin-Producing Cells

**DOI:** 10.4274/Jcrpe.1284

**Published:** 2014-06-05

**Authors:** Jessica Walter, Ole Harder, Fred Faendrich, Maren Schulze

**Affiliations:** 1 Schleswig Holsetin University Hospital, Campus Kiel, Department of General, Thoracic, Transplantation and Pediatric Surgery, Kiel, Germany; 2 University Hospital Essen, Department of General, Visceral and Transplantation Surgery, Essen, Germany

**Keywords:** Diabetes, autologous cell therapy, peripheral blood, human monocytes

## Abstract

**Ob­jec­ti­ve:** The vision of potential autologous cell therapy for the cure of diabetes encourages ongoing research. According to a previously published protocol for the generation of insulin-producing cells from human monocytes, we analyzed whether the addition of growth factors could increase insulin production. This protocol was then transferred to a non-human primate model by using either blood- or spleen-derived monocytes.

**Methods:** Human monocytes were treated to dedifferentiate into programmable cells of monocytic origin (PCMO). In addition to the published protocol, PCMOs were then treated with either activin A, betacellulin, exendin 3 or 4. Cells were characterized by protein expression of insulin, Pdx-1, C-peptide and Glut-2. After identifying the optimal protocol, monocytes from baboon blood were isolated and the procedure was repeated. Spleen monocytes following splenectomy of a live baboon were differentiated and analyzed in the same manner and calculated in number and volume.

**Results:** Insulin content of human cells was highest when cells were treated with activin A and their insulin content was 13 000 µU/1 million cells. Insulin-producing cells form primate monocytes could successfully be generated despite using human growth factors and serum. Expression of insulin, Pdx-1, C-peptide and Glut-2 was comparable to that of human neo-islets. Total insulin content of activin A-treated baboon monocytes was 16 000 µU/1 million cells.

**Conclusion:** We were able to show that insulin-producing cells can be generated from baboon monocytes with human growth factors. The amount generated from one spleen could be enough to cure a baboon from experimentally induced diabetes in an autologous cell transplant setting.

## INTRODUCTION

It is estimated that today, over 1 million individuals currently are diagnosed with type 1 diabetes mellitus (T1DM) in the USA. Due to the high profile of diabetes and the implementation of new genetic screening programs for families and newborns, the actual incidence of T1DM appears to be increasing (1,2).

Both the Diabetes Control and Complication Trial and the UK Prospective Diabetes Study Group demonstrated a strong correlation between good metabolic control and the rate/progression of complications in diabetic patients (3,4). The only present means of curing T1DM is through replacement of the pancreatic islet beta cells with either an artificial pancreas or glucose-responsive insulin-producing tissue. Currently, whole-pancreas organ transplantation and islet transplantation are considered the best chances for a cure. Other therapeutic options that are currently in the stage of preclinical investigations include immune cell ablation followed by an allogeneic bone marrow or hematopoietic stem cell transplant (5) and a cell-based treatment option to combat autoimmunity and restore beta-cell function (6). Both options may offer individuals who are genetically at risk a therapeutic intervention prior to onset of disease.

While ecto-pancreatic transplantation of donor pancreas has proven efficient in normalizing blood glucose levels, hemoglobin A1c , secretion of insulin and C-peptide, recent studies by Shapiro et al (7,8,9,10,11), showing impressive results in reversing T1DM following islet implantations in patients with severe metabolic instability, have focused attention on this intervention strategy. However, the success of this protocol underscores an already acute shortage of implantable islets. Thus, it is imperative to identify new sources of endocrine pancreas or surrogate tissue.

Monocytes have also been used by our group (12) and by Hur et al (13) to generate insulin-producing cells. Our protocol includes growth factors treatment for subsequently undergoing dedifferentiation followed by programmability (12). We have previously shown that these programmable cells of monocytic origin (PCMO) can then differentiate into insulin-producing cells (14) and hepatocyte-like cells (neo-hepatocytes) (15). PCMO derived insulin-producing cells have been shown to successfully normalize blood glucose levels in streptozotocin-treated mice (14). However, this effect was only seen over a period of 10 days. Furthermore, PCMO-derived insulin-producing cells did not produce sufficient amounts of insulin to theoretically treat a human being.

As the embryonic development of the pancreas shows us (16,17), members of the TGF-ß family and GLP-1-like proteins influence the endocrine differentiation of pancreatic progenitor cells into beta-cells. Factors that are promising include activin A (18,19), betacellulin (20) and the exendins 3 and 4 (21,22,23).

Most models describing the potential cure of diabetes by any means of cell therapy work with rodent animal models (24). On the other hand, a further step towards proof of concept in a human being would be the successful treatment of a large animal and preferentially, a non-human primate. At this stage, the handling of monkey cells is widely unknown as there are only a few protocols describing the differentiation of monkey embryonic stem cells (ESC) or adult monkey stem cells successfully being differentiated into insulin-producing cells (25,26,27). Hirshberg et al (28), described on of the few in vivo models of pancreatic islet transplantation into non human primates.

After identification of an optimized protocol for the generation of PCMO-derived insulin-producing cells, we applied this protocol on monocytes from non-human primates, in preparation for a non-human primate model of induced diabetes with potential autologous application of monocyte-derived insulin-producing cells.

## METHODS

**Isolation, Purification and in vitro **

**Culture of Monocytes**

PCMOs were generated from human peripheral blood monocytes following the protocol of Ruhnke et al (14). In brief, mononuclear cells from heparinized blood, buffy coats, or leukoreduction system chambers were isolated by density gradient centrifugation (Ficoll-Paque, Amersham Pharmacia Biotech AB, Uppsala, Sweden). Cells were cultured in either 24-well or 6-well plates (Cell+, Sarstedt, Nümbrecht, Germany) for various lengths of time in PCMO medium [Roswell Park Memorial Institute (RPMI) 1640 medium (Invitrogen, Karlsruhe, Germany)], supplemented with 5 ng/mL final concentration of M-CSF and 0.4 ng/mL final concentration of IL-3 (both from R&D Systems, Wiesbaden, Germany), 90 μM 2-mercaptoethanol and 10% human AB serum (Lonza, Verbier, Belgium). 1 hour after plating, cultures were gently washed to enrich for adherent cells and fresh medium was added to the adherent cell layer resulting in enrichment of 60%-70% CD14+ cells.

**Splenectomy and Isolation of Spleen Monocytes**

Ethics committee approval was issued from the Animal Research Ethics Committee, Health Science Faculty, University of Cape Town. Baboons (Papio Anubis) were held in a 12-hour day/12-hour night schedule in single cages with sight contact to others. Animal care was provided through veterinary visits on a regular schedule and daily care by qualified animal keepers.

The baboons were sedated by intramuscular injection of ketamin and transported to the operating room. After endotracheal intubation, general anaesthesia was achieved by isoflurane inhalation and intravenous administration of 2x5 mg morphine. After disinfection, a midline incision was performed and muscular sheaths were divided. The spleen was mobilized from the left retroperitoneum. The splenic artery and vein were ligated under visualization of the pancreatic tail. After removal of the spleen, the small bleeding sites were coagulated and the abdomen was closed. Temgesic 0.3 mg was administered IV for postoperative analgesia.

Under laminar air flow, the spleen was dissected and the capsule removed. The parenchyma was homogenised and diluted, then filtered through a mesh. This spleen solution was then treated as blood or buffy coat.

**Generation of Insulin-Producing Cells**

PCMOs were further cultivated as previously described in an RPMI 1640-based medium in addition of 10 ng/mL EGF, 20 ng/mL HGF (Calbiochem, Munich) 10 mM Nicotinamide (Sigma, Munich) and 5 mM glucose (14) for 7 days. As we have previously only induced suboptimal insulin production, the cells were additionally cultivated with addition of three different growth factors A: Activin A (2 ng/mL) (R&D Systems, Wiesbaden), B: Betacellulin (0.5 ng/mL) (Sigma Aldrich, Taufkirchen, Germany), C: Exendin 3 (10 nmol/mL) (Sigma Aldrich, Taufkirchen, Germany) and D: Exendin 4 (10 nmol/mL) (Sigma Aldrich, Taufkirchen, Germany). Non-human primates PCMO were only treated with supplement A.

**Immunohistochemistry**

PCMOs from non-human primate monocytes were characterized and identified by immunohistochemistry with primary antibodies listed in Table 1. Cells were harvested and washed and then cytospin were prepared. Several techniques of conventional immunohistochemistry such as Apaap (29) and avidin biotin (30) were used. Insulin-producing cells were equally stained as cytospin preparation against primary antibodies listed in Table 2. Primary antibodies were visualized by immune fluorescence technique (31) either as single or double staining.

**Elisa**

1x10^6^ cells were harvested, washed, lysed by ultrasound and resuspended in 1 mM acetic acid (Merck, Darmstadt, Germany) with 0.1% BSA. Insulin content was measured with an ELISA KIT (INS-EASIA, Cat.-No.: 4012500, Biosource, Nivelles, Belgium) according to the manufacturer’s instructions.

Absorption was measured at 450 and 490 nm against a reference wave length of 650 nm by a Thermo Max Microplate Reader (MWG Biotech). The data were analyzed with MikroWin software Version 3.0 (Mikrotek, Overath). Target antigen for the ELISA was human insulin with a cross reactivity to porcine insulin.

**Dithizone Uptake**

Insulin-producing beta cells contain a high amount of zinc (Zn2+). Dithizone is a chelating agent that binds to zinc and stains insulin-producing cells specifically by binding to zinc-insulin complexes. Cytospin containing insulin-producing cells derived from non-human primate PCMO’s were incubated for 5 minutes in a solution of 10 mg Dithizone (ICN Biomedicals, Eschwege, Germany) in 1 mL DMSO (Dimethylsulfoxid, WAK-Chemie Medical, Steinbach, Germany) and 9 mL HBSS (Hank’s Buffered Salt Solution, Lonza, Walkersville, USA), that was filtrated prior to use. 

## RESULTS

**Optimizing Insulin Production in Human **

**PCMO-Derived Insulin-Producing Cells**

**Figure 1:**
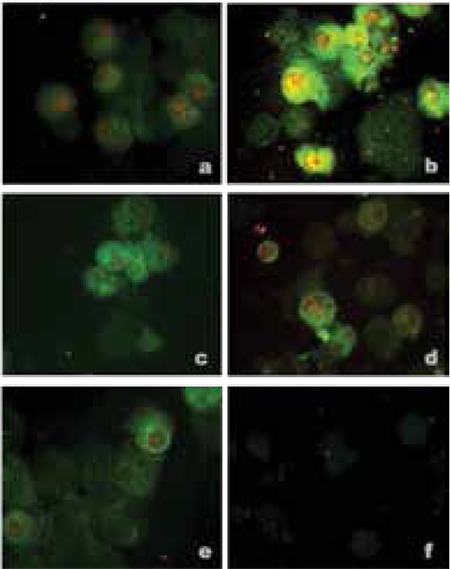
a-f. Immunohistochemical staining of human PCMO-derived insulin-producing cells for insulin and Pdx-1. a) Medium without supplement; b) Activin A; c) Betacellulin; d) Exendin 3; e) Exendin 4; f) Negative control

The addition of betacellulin, exendin 3 and exendin 4 to the standard medium containing HGF, EGF and nicotinamide only revealed a moderate increase in insulin and Pdx-1 expression (Figure 1 a,c,d,f,e). However, activin A-treated cells showed a marked increase in insulin expression on the immunohistochemical staining (Figure 1b). 

**Figure 2:**
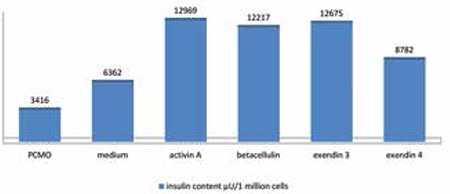
Insulin content of 1 million human PCMO cells alone or after cultivation with additional growth factors (activin A, betacellulin, exendin 3 or exendin 4) was measured using ELISA technique

Insulin content in 1 million lysed cells showed an increase in activin A-treated cells as compared to standard culture medium and the three other test media containing betacellulin, exendin 3 and exendin 4 (Figure 2).

**Characterization of PCMOs from Non-Human Primates**

**Figure 3:**
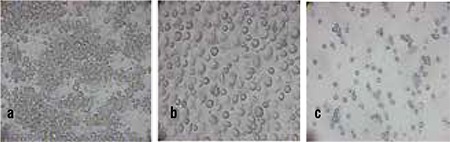
a, b,c. a) PCMO from non-human primates 6 days after culture; b) Cells cultures without MCSF and Il3 differentiated into macrophages; c) Apoptosis after a prolonged dedifferentiation period

**Figure 4:**
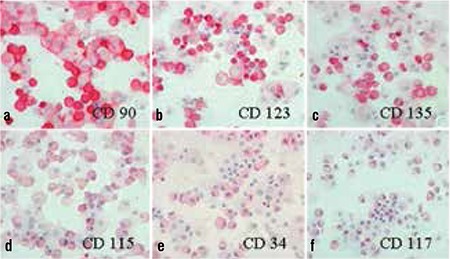
a-f. Immunohistochemical APAAP staining. a) CD90; b) CD 123; c) CD135; d) CD115; e) CD34; f) CD117

After the six-day culture period with MCSF and Il-3 supplemented medium, baboon blood- or spleen-derived monocytes acquired the characteristic morphology as described for human cells (Figure 3a). Cells cultured without the presence of MCSF and Il-3 differentiated into macrophages (Figure 3b). After a prolonged dedifferentiation period, the cells underwent apoptosis (Figure 3c). Immunhistochemical staining showed an upregulation of PCMO characteristic markers CD90, CD123 and CD135 (Figure 4 a,b,c). The MCSF receptor antigen, CD115, was expressed until day 6, in declining intensity (Figure 4d). Classical hematopoietic stem cell markers (CD34 und CD117) were only weakly expressed during the entire culture period (Figure 4 e,f).

**Generation of Insulin-Producing Cells from **

**Non-Human Primate PCMO Morphology and **

**Dithizone Uptake**

**Figure 5:**
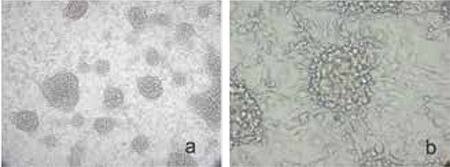
a,b. a) Morphology of non-human primate PCMO derived insulin-producing cells showing characteristic cluster; b) Morphology of non-human primate PCMO showing characteristic neuron-like processes

**Figure 6:**
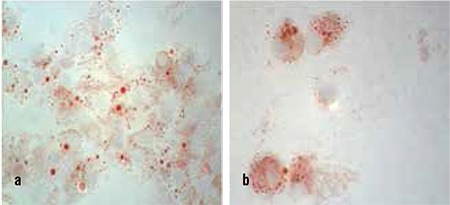
a,b. a) Dithizone staining of non-human primate insulin-producing cells showing the typical distribution pattern for insulin uptake; b) Dithizone staining in high magnification with vesicular staining

PCMOs from non-human primate monocytes were exclusively cultured further in standard medium containing HGF, EGF and nicotinamide with addition of only activin A. The morphology of these cells after 7 days’ treatment was very characteristic for insulin-producing cells as known from human monocyte-derived cells (Figure 5a). Cells aggregated in three dimensional clusters with neuron-like processes (Figure 5b). Incubation in dithizone showed an uptake of the zinc-chelating dye in red intracellular granules (Figure 6 a,b) suggesting insulin content.

**Protein Expression and Insulin Content **

**Figure 7:**
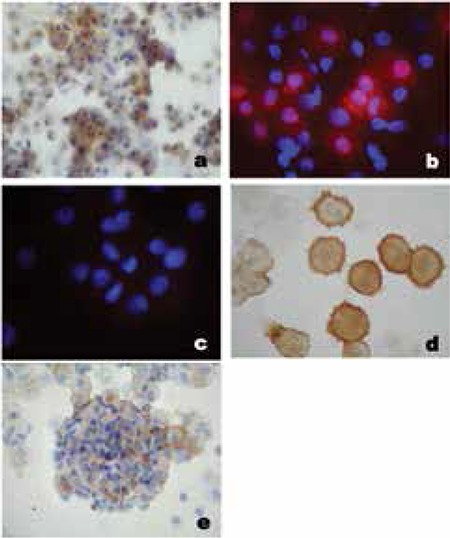
a-e. Staining of non-human primate insulin-producing cells: a) Insulin in perinuclear localization; b) Staining against C-peptide; c) Negative control; d) Glut-2 on the cell membrane; e) Pdx-1

**Figure 8:**
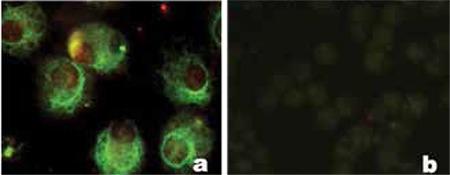
a,b. a) Double immunohistochemistry for insulin and Pdx-1; b) Negative control

**Figure 9:**
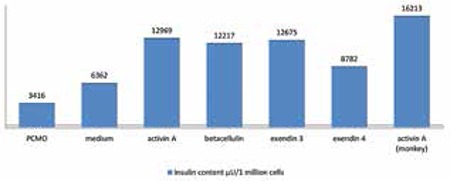
Insulin content of human PCMO cells alone or with addition of growth factors (as shown in Figure 2) is compared to insulin content of 1 million non-human primate derived PCMO cells treated with activin A, showing a higher insulin content in NHP derived cells, measured with ELISA

Visualization of insulin in non-human primate monocyte-derived cell clusters showed a typical perinuclear staining pattern (Figure 7a). Staining against the C-peptide antigen confirmed de novo insulin synthesis (Figure 7b). Figure 7c shows negative control of the cells without primary antibody. The glucose receptor Glut-2 could be visualized on the cell membrane (Figure 7d). The transcription factor Pdx-1 necessary for the insulin production pathway however was only expressed in a subpopulation of the cells arranged in clusters (Figure 7e). Double immunohistochemistry showed a simultaneous localization of insulin and Pdx-1 in the cell cytoplasma (Figure 8 a,b). Insulin content in these activin A-treated non-human primate monocyte-derived cells was measured in 1 million lysed cells after 12-hour incubation in 5 mmol glucose-containing medium. An insulin content of 16 213 µU/1 million cells was comparable to that of human cells treated with activin A (Figure 9).

**Total Splenectomy as a Cell Source in an Autologous**

**Cell Therapy Model for Non-Human Primates**

Splenectomy of a baboon yielded 4x108 insulin-producing cells. Based on a calculation according to the Edmonton protocol which uses 1x104 cells per kg bodyweight, this is more than enough for an autologous treatment of a 40-kg animal (32). According to this calculation, we would need only 4x10^6^ insulin-producing cells for the treatment of a 40-kg baboon.

Postoperative recovery of the animals after total splenectomy was successful, with primary scar healing and no signs of immune deficiency.

## DISCUSSION

In this study, an increased insulin production and expression of Pdx-1 of PCMO-derived insulin-producing cells was found after supplementation of activin A to the differentiation medium. Furthermore, the protocol could be applied to a non-human primate model using baboons, in which a sufficient mass of cells could be generated by differentiation of spleen-derived monocytes through splenectomy.

There is growing evidence over the important role of activin A in development of pancreatic differentiation. Activin A is a member of the TGF-ß family and interacts with two types of cell surface transmembrane receptors, which both have intrinsic serine/threonine kinase activities in their cytoplasmic domains. Binding to the type II receptor initiates a cascade reaction of recruitment, phosphorylation and activation of type I activin receptors. This leads to a phosphorylation of SMADs2/3 followed by translocation of SMAD3 to the nucleus, interaction with SMAD4 through multimerization, resulting in activation of transcription factor complexes which are responsible for specific gene expression (18). Furthermore, activin A has been shown to induce ESC into pancreatic and neural lineages (32). D‘Amour et al (33) were able to demonstrate a significant influence of activin A in the development of hormone-expressing endocrine cells throughout definitive endoderm. Accordingly, several studies have shown this promotion of definitive endoderm differentiation of both mouse (34,35) and human ESC (hESC) (36) by administration of activin A. These differentiated cells however seemed to be only a precursor of definitive insulin-producing cells, due to the fact that the cells showed expression of additional hormones like glucagon and showed no glucose-dependant insulin secretion. In addition, activin A was addressed to play an important role in the maintenance of the pluripotency of hESC (37,38) underlining the complexity of pathways in their differentiation. Refined culture protocols for inducing pancreas-committed cells from hESC has been reported by Xu et al (39), demonstrating an interplay between activin A, BMP and FGF which seemed to induce further differentiation steps.

In contrast to the mentioned studies in which hESC were the cell source of interest, we worked with cells of monocyte origin, i.e. PCMOs, which were introduced in recent publications (14). We are aware of only one group of investigators (13) who described the differentiation of human peripheral blood monocytes to insulin-producing cells. These investigators showed that monocytes can be differentiated into insulin-producing cells by hematosphere culture. They showed in vitro insulin production but have not reported any evidence showing in vivo normalization of blood glucose levels. The main advantages of monocyte-derived cells over hESC are the possibility of easy access to these cells and absence of teratogenic effects.

PCMOs were differentiated into neo-islet cells which were found to induce transient glucose normalization after transplantation in diabetic mice. This temporary effect is most likely due to a precursor stage of ß-cells. In the current study, we were able to refine the differentiation protocol for human PCMO by adding activin A to the medium, leading to an increase of insulin content of 213% compared to the medium without activin A. Nevertheless, the insulin content is still less than that of an adult ß-cell, indicating the lasting distance to a full differentiation. A more complex environment including extracellular matrix and intercellular interactions may be needed, which is hard to mimic in an in vitro environment. 

The main achievement of this study was the transfer of the protocol into a non-human primate model. Blood- and spleen-derived monocytes from non-human primates could be isolated and successfully differentiated to PCMO. The characterization was done by the characteristic morphology and immunhistochemical staining with human antibodies.

The use of non-human primate models provides a tremendous benefit in the investigation of diabetes. Recently, the use of rhesus monkeys in pancreatic islet transplantation studies provided critical information that proved to be helpful in the modification of the islet transplant protocol (13). The literature contains only very few non-human primate models, which might be due to practical difficulties in differentiation of non-human primate cells, such as the unavailability of species-specific antibodies. Regardless, Lester et al (26) investigated the differentiation of rhesus monkey ESC to insulin-producing cells ex vivo.

To our knowledge, we are the first group that showed a differentiation of non-human primate PCMOs to insulin-producing cells. The non-human primate PCMOs were cultured in the activin A-supplemented differentiation medium and the resulting insulin content was comparable to that in human insulin-producing cells. In our view, this is a promising baseline for further studies in the primate model. Splenectomy was performed uneventfully and achieved a sufficient cell mass for further transplantation studies, based on the calculation of the Edmonton protocol. The resulting cell mass of one monkey spleen would be enough to perform autologous cell transplantation after differentiation of the PCMO to insulin-producing cells and induction of diabetes in the monkey. 

In conclusion, in this study, we were able to show that activin A increases the insulin production of insulin-producing cells derived from PCMO. Furthermore, PCMO could be generated from non-human primate blood and spleen and then be differentiated to insulin-producing cells by our protocol. With the proof of a sufficient cell yield after uneventful splenectomy, these results mark the basis for further investigations in the diabetic non-human primate model.

## Figures and Tables

**Table 1:**
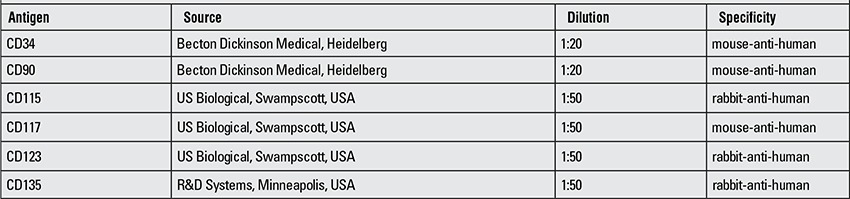
Primary antibodies for immunohistochemistry staining of programmable cells of monocytic origin from non-human primate monocytes

**Table 2:**
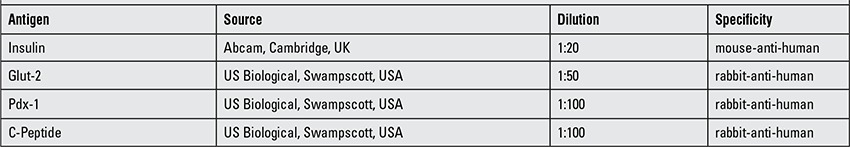
Primary antibodies for staining of insulin-producing cells
